# Influence of discordant tubal blockage on clinical pregnancy rates: a retrospective cohort study

**DOI:** 10.1007/s00404-025-08186-1

**Published:** 2025-09-23

**Authors:** Franziska Elisabeth Bormann, Marlene Hager, Sophie Luise Thieme, John Preston Parry, Johannes Ott

**Affiliations:** 1 Karl Landsteiner University of Health Sciences, Dr.-Karl-Dorrek-Straße 30, 3500 Krems, Austria; 2 Clinical Division of Gynecological Endocrinology and Reproductive Medicine, Medical University of Vienna, Spitalgasse 23, 1090 Vienna, Austria; 3 Parryscope and Positive Steps Fertility, Madison, Madison, WI 39110 USA; 4 Department of Obstetrics and Gynecology, University of Mississippi Medical Center, Jackson, MS 39216 USA

**Keywords:** Fallopian tubes, Chromopertubation, Laparoscopy, Female infertility, Pregnancy rate, Discordant tubal blockage

## Abstract

**Purpose:**

To present recent data on discordant tubal blockage (DTB), its influence on pregnancy rates and how women should gage their fertility when screening and diagnostic tests don’t always agree.

**Methods:**

This retrospective cohort study included 78 infertile women, who underwent tubal patency assessment between January 2016 and June 2024 at the Clinical Division of Gynecological Endocrinology and Reproductive Medicine, Medical University of Vienna. Tubal patency was assessed twice. Initial assessment of tubal patency had been performed by hysterosalpingo-contrast sonography (HyCoSy) or hysterosalpingography (HSG) and had suggested bilateral occlusion in the DTB group (*n* = 38) and bilateral patency in controls (*n* = 38). Bilateral patency was found in all patients during subsequent laparoscopic chromopertubation. The primary outcome parameter was the clinical pregnancy rate within 6 months.

**Results:**

The basic patient characteristics showed no significant differences between the DTB and the control groups. Clinical pregnancy was found in 47.4% (18/38) of control patients (patent tubes) and in 21.1% (8/38) of DTB patients (*p* = 0.029) over 6 months of follow-ups. In a multivariate model, younger age [odds ratio (OR), 0.856, *p* = 0.013] and bilateral patency (OR 4.210, *p* = 0.010) in both examinations (control group) were associated with higher clinical pregnancy rates.

**Conclusion:**

DTB reflects lower fecundity even when subsequent patency is demonstrated. Tubal patency following prior occlusion should not be grounds for complete reassurance, and given lower odds of pregnancy, such patients may be warranted a faster transition to ART given the decreased efficacy of other methods.

## What does this study add to the clinical work?

**Table:** 

Tubal patency following prior occlusion should not be grounds for complete reassurance, and given lower odds of pregnancy, such patients may be warranted a faster transition to ART given the decreased efficacy of other methods.	

## Introduction

In 2023, the WHO acknowledged that one in six reproductive adults will experience infertility [1]. This high prevalence reflects its relevance and speaks to the need for effective procreative care. Tubal factor infertility (TFI) plays a role in 25% of infertile women according to recent data [2], which is in line with the prevalence of 25–30% reported by a study from 1989 [3]. Common methods of assessing tubal patency include hysterosalpingography (HSG), hysterosalpingo-contrast sonography (HyCoSy), laparoscopic chromopertubation, and, increasingly, hysteroscopic assessment. HSG and HyCoSy are less-invasive techniques since no surgery is required. Laparoscopy with chromopertubation is considered the gold standard, but given the typical effectiveness of screening methods, it is primarily performed for superimposed additional indications.

The impact of contradictory results between screening and diagnostic tests on fecundity was previously assessed by Gleicher et al. [4], leaving data on this issue sparse. Though typically described as tubal spasm, technically this is primarily myometrial contraction around the tube even though the tube does have muscles [5]. Such spasms have been documented during both HSG [6] and laparoscopic chromopertubation [7]. Notably, pain with dilation and application of the contrast medium/blue dye can amplify myometrial spasm, which explains why patients with pelvic pathology (adhesions, endometriosis) are more susceptible to tenderness which can drive spasms even with gentle technique. Moreover, chlamydia cervicitis structuring the cervix, coupled with tubal stricture associated with previous infection, can make for more painful introduction of media given that entry requires a high pressure due to difficult egression (through occlusion of outflow). This means that while treatment can have false positives, a previous infection can increase the risk for a spasm, suggesting full occlusion which may not be present, but also being a proxy for intraluminal damage. To enlarge our study population, we decided to compare patients with patent tubes according to HSG/HyCoSy and chromopertubation with patients showing signs for DTB after HSG/HyCoSy and chromopertubation.

Doctors at the Medical University of Vienna reported cases with subjectively elevated flushing pressures during chromopertubation. This phenomenon was also described by a study in 2004 [8], which strengthens the significance of elevated flushing pressure, probably being associated with poor pregnancy rates. Recent reliable data remain limited even though discordance between screening and diagnostic testing remains an important issue. Our retrospective study further explores how discordant findings impact fecundity.

## Methods

### Patient population

This retrospective study included 78 infertile women, ages 18–40, who underwent tubal patency testing via HSG or HyCoSy, followed by laparoscopic chromopertubation at the Clinical Division of Gynecologic Endocrinology and Reproductive Medicine, Medical University of Vienna, Austria, between January 2016 and June 2024. In women of Group A, HSG or HyCoSy demonstrated bilateral tubal patency, which was confirmed by laparoscopic chromopertubation subsequently. In women of Group B, contradictory results were identified: bilateral tubal occlusion had been diagnosed using HSG or HyCoSy, whereas subsequent laparoscopic chromopertubation showed bilateral tubal patency.

Women underwent laparoscopy for the following indications: suspicion of tubal occlusion (Group B); suspicion of endometriosis; presence of ovarian cysts in ultrasound; and unexplained infertility. Hysteroscopy was performed in addition as part of the routine procedure in this patient population.

The following exclusion criteria were applied: uni- or bilateral hydrosalpinx; uterine fibroids; polycystic ovary syndrome (PCOS); oligo- or amenorrhea, shortened menstrual cycles < 24 days; ASRM stage 3 and 4 endometriosis; previous unilateral or bilateral salpingectomy; planned intrauterine insemination or IVF or ovarian stimulation/ovulation induction; women with partners with abnormal semen analysis results. Notably, all women were advised to start trying to conceive subsequent to the operation. All patients revealed regular cycles (length 26–30 days) and were counseled to use ovulation tests for self-monitoring of their cycle. In case of a pregnancy or after 6 months, patients were called in for re-evaluation.

This study was approved by the Ethics Committee of the Medical University of Vienna on July 7th, 2023 (IRB number: 1395/2023).

### Sample size analysis

The previous study by Gleicher et al. showed a 10-percentage-point difference in pregnancy rates [4]. However, there is a significant difference between the design of their study and ours: In our research, the control group consisted of women with consistently patent tubes, whereas Gleicher’s control group included patients who underwent tubal cannulation after occlusion. We assumed that women with consistently patent tubes would reveal a higher chance to conceive naturally and, thus, assumed an absolute difference of 30% between the two groups for clinical pregnancy rates within 6 months after the operation (40% in Group A, 10% in Group B), which was also based on unpublished data of our study group. Based on this information, a sample size of 38 patients per group was necessary to achieve statistical significance at *p* = 0.05, with a power of 80% after correction of Fleiss.

### Parameters analyzed

Data acquisition was conducted using AKIM Software (version 7, SAP Software Solutions Austria, Vienna, Austria; SAP-based patient management system at the Medical University of Vienna). The main outcome parameter was clinical pregnancy after 6 months of regular, unprotected sexual intercourse. In addition, we also included the following data: results of tubal patency testing; age at surgery; body mass index (BMI); infertility type (primary versus secondary); indication for laparoscopy (preliminary concern for tubal occlusion, suspicion of endometriosis, presence of ovarian cysts, unexplained infertility); and whether endometriosis was diagnosed using laparoscopy [2].

### Assessment of tubal patency

The following techniques were used to assess tubal patency for the patients included in this study: HSG and HyCoSy were performed by experienced clinicians according to international standards [9, 10]. Laparoscopic chromopertubation was carried out as reported previously [2, 11, 12]. The surgical assessment of tubal patency was performed under general anesthesia, and either executed or supervised by experienced specialists in infertility surgery. For this procedure, a Spackman uterine manipulator with clamp fixation and a rubber cone (18 mm diameter) (reference number 1264; WISAPR Medical Technology GmbH, Brunnthal/Hofolding, Brunnthal, Germany) was inserted through the cervix, positioning the tip one centimeter from the uterine fundus. Chromopertubation was conducted using a syringe containing 50 mL of a diluted indigo carmine blue dye solution (Amino AG, Gebenstorf, Switzerland) to assess tubal patency. All women included with endometriosis revealed superficial peritoneal lesions only, and endometriosis was removed completely in all cases.

### Statistical analysis

Statistical analysis was performed with the SPSS software package, version 28.0.1.0 (SPSS, Chicago). Numerical variables are reported as median and interquartile ranges (IQR), and categorical variables as numbers (frequency). Groups were compared using analysis of variances (ANOVA) for numerical parameters and Fisher’s exact tests for categorical parameters. To test factors associated with the chance for a clinical pregnancy, univariable, followed by multivariable, binary logistic regression models were used. Only parameters which were statistically significant in the univariable approach were entered into the multivariable model. This analysis used odds ratios (OR) with 95% confidence intervals (95% CI). Differences were considered significant if *p* < 0.05.

## Results

An overview of basic patient characteristics, which also include the indications for laparoscopy and the final diagnosis of endometriosis, is provided for both study groups in Table 1. No significant differences were found. Notably, endometriosis was detected laparoscopically in 44.7% of Group A patients and 42.1% of Group B patients. In the minority of women, the fertility was secondary (17/76, 22.4%). Of these, 13 women had suffered from one or two early miscarriages, whereas four patients (2 in each study group) had given birth to one child.

**Table 1:** Basic characteristics of group A and group B patients

	Group A (*n* = 38)	Group B (*n* = 38)	*p*
Age (years)^1^	31.0 (28.5; 33.3)	31.4 (22.8; 33.2)	0.662
BMI (kg/m^2^)^1^	22.7 (19.9; 26.8)	22.5 (20.0; 26.4)	0.776
Primary infertility^2^	28 (73.7)	31 (81.6)	0.583
Indications for laparoscopy^3^			
Preliminary concern for tubal occlusion^2^	0	38 (100)	< 0.001
Suspicion of endometriosis^2^	20 (52.6)	20 (52.6)	1.000
Ovarian cyst^2^	8 (21.1)	15 (39.5)	0.133
Unexplained infertility	10 (26.3)	0	0.025
Final diagnosis of endometriosis^2^	17 (44.7)	16 (42.1)	1.000

Data are provided as ^1^median (IQR) for numeric parameters or ^2^*n* (%) for categorical parameters, ^3^multiple mentions possible

After 6 months of regular menstrual cycles, clinical pregnancy was found more often in women of Group A (bilateral tubal patency in both HSG/HyCoSy and chromopertubation: 18/38, 47.4%) than in Group B patients (bilateral tubal occlusion in HSG/HyCoSy followed by bilateral tubal patency in chromopertubation: 8/38, 21.1%; *p* = 0.029). Predictive factors for clinical pregnancy were tested in a univariate, followed by a multivariate, binary logistic regression model (Table 2). In both models, a lower age (OR 0.877, *p* = 0.024 and OR 0.856, *p* = 0.013, respectively) and Group A (OR 3.375, *p* = 0.018 and OR 4.210, *p* = 0.010, respectively) were significantly associated with a higher chance for clinical pregnancy.

**Table 2:** Predictive factors for clinical pregnancy: results of a univariable flowed by a multivariable regression model

	Clinical pregnancy (*n* = 26)	No clinical pregnancy (*n* = 50)	Univariable model		Multivariable model	
			OR (95% CI)	*p*	OR (95% CI)	*p*
Age (years)^1^	29.2 (26.8; 31.6)	32.0 (28.6; 34.0)	0.877 (0.783; 0.983)	0.024	0.856 (0.758; 0.967)	0.013
BMI (kg/m^2^)^1^	22.7 (19.3; 27.4)	22.5 (20.5; 26.2)	1.016 (0.915; 1.128)	0.770	–	–
Primary infertility^2^	21 (80.8)	38 (76.0)	1.326 (0.411; 4.280)	0.637	–	–
Endometriosis^2^	9 (34.6)	24 (48.0)	0.574 (0.215; 1.528)	0.266	–	–
Group A^2^	18 (69.2)	20 (40.0)	3.375 (1.233; 9.237)	0.018	4.210 (1.412; 12.556)	0.010

Data are provided as ^1^median (IQR) for numeric parameters or ^2^*n* (%) for categorical parameters

## Discussion

These research findings fundamentally call into question the widespread practice of telling patients that they are fine and it was simply tubal spasm if they have tubal occlusion at screening, followed by patency at laparoscopy. This study found significant differences between the two cohorts in the likelihood of achieving natural pregnancy after a 6-month follow-up period. Patients with consistently patent fallopian tubes in both preoperative HyCoSy/HSG and laparoscopic chromopertubation yielded higher pregnancy rates compared to patients with DTB. While this finding may seem intuitive, it has not been thoroughly examined in previous research. DTB may represent a substantially compromised form of tubal patency, negatively affecting fertility. The impact of DTB on pregnancy rates was particularly striking as women with patent tubes had a pregnancy rate of 47.4% (18/38), more than twice as high as the 21.1% (8/38) observed in the DTB group.

Though the magnitude of difference with a short-term follow-up is striking, in some ways, the findings should not be. The concept of tubal disease as “patent” or “non-patent” is reductive and oversimplifying. Rather than as a pipe, as so many describe the Fallopian tubes, a conveyor belt is a more apt analogy given the ciliary function. We have previously shown that inflammatory states such as endometritis increase intraluminal damage, as well as that up to 95% of occlusion is proximal or full length and not just purely distal [11]. Cannulation and more aggressive measures cannot restore ciliary function when repairing intrinsic and not just extrinsic damage (peritubal adhesions and phimosis). Moreover, if under anesthesia at laparoscopy, one can use greater supraphysiologic pressures for chromopertubation than a conscious patient would tolerate with a screening test, technical patency does not necessarily reflect a normal tube or a normal fecundity. Accordingly, we need to shift the paradigm for when occlusion is suggested at HSG or HyCoSy from one of the open or the closed, instead to one of the proxies for the degree of risk of tubal inefficiency lowering fecundity with spontaneous or non-ART-assisted conception.

Another factor significantly associated with lower pregnancy rates was younger age. Younger women had a higher chance of becoming pregnant within 6 months of follow-ups. The median age of women who conceived was 29.2 years, compared to 32.0 years in those who did not. In a univariable model, the *p*-value was 0.024, while in a multivariable model, it was 0.013, indicating even greater relevance. The essence of this finding is not new, but it is reassuring that the study reaffirmed core biologic principles, where earlier in the reproductive years, there is higher fecundity, consistent with a lower probability of age-associated aneuploidy [13, 14].

A key objective of our study was to compare our findings with those of Gleicher et al., whose study served as the motivation for our retrospective analysis. Gleicher reported an absolute 10-percentage-point difference in pregnancy rates (39% vs. 29%) between their study groups [4]. However, beyond their having a longer duration of follow-ups, a methodological distinction must be made between the two studies. In our research, the control group consisted of women with consistently patent tubes, whereas Gleicher’s control group included patients who had undergone tubal cannulation after occlusion. This calls for attention to two core issues relating to tubal “occlusion” on screening and cannulation. The first is that cannulation does not “repair the hair”, where ciliary damage will persist even with successful cannulation. This likely explains why there was only a subtle increase in fecundity when cannulation was successfully achieved. Second, the yield of cannulation might be less dependent on inherent techniques, but rather on the probability of inaccuracy with the initial screening test. High success with cannulation could be more likely attributable to higher rates of spasms. When HSG is performed with suboptimal techniques, subsequent fertility could be based on normal tubes and not improved through cannulation per se (Parry, personal communication).

There are several core reasons why misleading occlusion at HSG may be associated with lower fecundity despite technical patency. As noted before, patency does not automatically translate to ciliary function. Moreover, pelvic sensitivity to pain may reflect a predisposition to inflammatory states, such as endometriosis, pelvic inflammatory disease-associated adhesions, endometritis, adhesions after ruptured appendicitis, and more. Accordingly, pain-induced uterine spasms occluding the tubes may be a proxy for pathology, independently creating a risk for subfertility. Moreover, though contrast dye and air bubbles can fit through a narrow tubal lumen, if there is stricture from previous damage, this could require more force to demonstrate patency, resulting in pain that, in turn, results in spasm again creating a false positive, suggestive of total occlusion instead of partial. All of these considerations call into question ultimately the yield of diagnostic laparoscopy when there is occlusion suggested by screening tests. Diagnostic laparoscopy may demonstrate technical patency, which matters, but fecundity may be greatly reduced even with patency. Given the costs and surgical delays inherent to laparoscopy, exploring assisted conception may offer a better balance than surgical confirmation to guide next steps.

Small sample size might be seen as a study limitation. Initially, we based our sample size calculation on the assumption of a 30-percentage-point difference in pregnancy rates between the groups (40% versus 10%). While the actual difference turned out to be slightly smaller, at 26 percentage points, it still represents a substantial and clinically meaningful gap. Even in the small multivariable analysis (Table 2), where the data were corrected for age, DTB remained a significant predictive parameter for pregnancy, with an OR of 4.210 (IQR 1.412–12.556; *p* = 0.010).

Notably, many women in both groups revealed other female factors for sub-/infertility, first and foremost endometriosis and ovarian cysts (Table 1), which explains the moderate pregnancy rate of 47% after 6 months. For comparison, a North American study from 2017 reported that women aged 31–33 years generally have a 61% chance of becoming pregnant within 6 months and 77% within 12 months [15]. According to European data from 2003, the pregnancy rate among truly fertile couples, using natural-family-planning applications, was 88% after 6 months of follow-ups and 98% after 12 months [16]. It can be seen as a potential limitation as we did not compare otherwise completely healthy DTB patients with the general fertile population. Empirically, many women who have bilateral tubal blockage in HyCoSy/HSG chose to undergo IVF directly and, thus, cases of DTB are quite rare, probably due to underdiagnosis. However, given the fact that the rates of endometriosis and ovarian cysts did not differ between the two groups (Table 1), this should only be a minor limitation. On a related note, many with DTB in our patient base had unilateral occlusion, which still matters, and while incorporating them into this study would have increased sample size, it would have made for less clean results.

It is also noteworthy that our DTB patients presented with tubal occlusion during the initial assessment (HyCoSy/HSG), whereas tubal patency was found during the second examination (chromopertubation). Had this sequence been reversed, the findings would have been less compelling in our view. This is because clinicians would intuitively interpret newly patent tubes following prior occlusion as a positive outcome, suggesting restored or intact tubal function. A transition from patency to occlusion, on the other hand, is more likely to raise doubts and prompt further diagnostic considerations. This is why our findings are particularly relevant: they help identify a subset of patients with tubal infertility that could otherwise be overlooked due to a seemingly favorable finding of tubal patency at the time of chromopertubation.

## Conclusion

In conclusion, our findings provide distinct insights into the detrimental impact of DTB on female fertility and emphasize the need for further research into the underlying mechanisms of tubal dysfunction. With a 26-percentage-point difference in pregnancy rates between DTB patients and the control group, we identified clinically relevant associations. The detection of tubal patency following a previous diagnosis of occlusion should be interpreted with caution. Although natural conception can occur in women with DTB, especially in younger women, patients should be informed that they could still be in need for IVF in future. A pregnancy rate of 21.1% over 6 months indicates pronounced below-average fertility [15]. Further studies are needed to uncover the causes and maybe develop potential treatments for DTB.

## Data Availability

Data are available from the authors upon reasonable request.
